# Prediction of nonsentinel lymph node metastasis in breast cancer patients based on machine learning

**DOI:** 10.1186/s12957-023-03109-3

**Published:** 2023-08-11

**Authors:** Yuting Xiu, Cong Jiang, Shiyuan Zhang, Xiao Yu, Kun Qiao, Yuanxi Huang

**Affiliations:** Department of Breast Surgery, Harbin Medical University Cancer Hospital, Harbin, 150086 China

**Keywords:** Machine learning, Nomogram, Breast cancer, Nonsentinel lymph node metastasis

## Abstract

**Background:**

Develop the best machine learning (ML) model to predict nonsentinel lymph node metastases (NSLNM) in breast cancer patients.

**Methods:**

From June 2016 to August 2022, 1005 breast cancer patients were included in this retrospective study. Univariate and multivariate analyses were performed using logistic regression. Six ML models were introduced, and their performance was compared.

**Results:**

NSLNM occurred in 338 (33.6%) of 1005 patients. The best ML model was XGBoost, whose average area under the curve (AUC) based on 10-fold cross-verification was 0.722. It performed better than the nomogram, which was based on logistic regression (AUC: 0.764 vs. 0.706).

**Conclusions:**

The ML model XGBoost can well predict NSLNM in breast cancer patients.

**Supplementary Information:**

The online version contains supplementary material available at 10.1186/s12957-023-03109-3.

## Introduction

Cancer and cardiovascular diseases are the two main causes of death across the world and seriously harm people’s physical and mental health [1]. According to data from the World Health Organization (WHO), the number of newly diagnosed cancers in 2020 totaled 19.29 million, of which 2.26 million were breast cancers, and approximately 685,000 died from breast cancer [2]. Breast cancer leads the world in morbidity and mortality rates in most countries [2]. At the same time, the treatment regimens of breast cancer are changing over time. In 1985, the results of the National Surgical Adjuvant Breast and Bowel Project (NSABP) B-06 study demonstrated that breast-conserving surgery combined with radiotherapy led to no significant difference in overall survival (OS) and disease-free survival (DFS) of patients with early breast cancer compared with mastectomy, which raised the proportion of breast cancer patients treated with breast-conserving surgery [3], and the safety of breast-conserving surgery was confirmed in the following 20 years of follow-up [4]. In 2010, the results of the NSABP B-32 study showed that for malignant breast tumor patients with negative axillary lymph nodes, the success rate of axillary sentinel lymph node biopsy (SLNB) was 97.2%, and the false-negative rate was only 9.8%. There were no significant differences in OS, DFS, or local recurrence rate (LRR) for patients with negative sentinel lymph nodes but without axillary lymph node dissection (ALND) compared with those who underwent ALND [5, 6]. The risk of lymphedema and reduced range of motion in the upper limbs associated with ALND is not negligible, and it seriously affects the quality of life of patients [7]. The AMAROS study showed that early breast cancer patients who underwent SLNB combined with radiotherapy had similar axillary lymph node recurrence and DFS rates as those who underwent ALND, even if there were 1 or 2 sentinel lymph node metastases (SLNMs) [8]. In 2015, the American College of Surgeons Oncology Group (ACOSOG) Z0011 study confirmed that SLNB combined with radiotherapy could exempt early breast cancer patients with 1 or 2 SLNMs from ALND [9, 10], which further promotes the clinical application of SLNB. However, ALND is required for breast-conserving surgery patients with more than three sentinel lymph node metastases or total mastectomy patients with more than one sentinel lymph node metastasis. Studies have shown that 40–60% of breast cancer patients who undergo SLNB and further undergo ALND have no other lymph node metastases [11–13]. With the progress of individualized treatment of breast cancer and patients’ increasing demand for quality of life, axillary lymph node management is more inclined to include the evaluation of tumor staging and prognosis to accurately predict the risk of axillary lymph node metastasis, which can avoid surgical complications caused by overtreatment and thereby improve patients’ quality of life. It can also help reduce the recurrence risk for breast cancer patients with nonsentinel lymph node metastases (NSLNMs) who undergo SLNB but not ALND.

In recent years, machine learning (ML) has been used to manage different medical problems, such as pathologic diagnosis and treatment support, and ML models constructed in previous studies not only have better model performance but also have higher prediction accuracy [14–16]. Few models have been constructed to predict NSLNM. Guo Xu and his team constructed a deep learning model to predict NSLNM, but they failed to explain the impacts of different variables in their model [17]. Yang, ZB et al. [18] developed a nomogram to predict NSLNM, which showed an area under the curve (AUC) of 0.718 in the training set and 0.742 in the validation set, but its performance had not been compared with that of ML models.

Lundberg et al. first conceived the SHapley Additive exPlanations (SHAP) framework, which has been applied to machine learning [19]. It can assess the contributions of different features in different ML models, allowing the performance of each model to be reasonably compared [20].

The purpose of this study was to construct an optimal ML model to predict the NSLNM of breast cancer patients by using preoperative and intraoperative clinicopathological and imaging features and to choose the best model by using the SHAP framework. This study also compared its performance with that of a nomogram.

## Materials and methods

### Patients

A total of 3658 malignant breast cancer patients undergoing surgery at Harbin Medical University Cancer Hospital from June 2016 to August 2022 were retrospectively enrolled. This study was approved by the Ethics Committee of Harbin Medical University Cancer Hospital. It conforms to the 1964 Helsinki Declaration of the World Medical Association and its subsequent revisions. Informed consent from our hospital was signed by each patient before receiving treatment.

The inclusion criteria were as follows: no other breast cancer treatment prior to breast surgery and SLNB and ALND performed during breast surgery.

Exclusion criteria are as follows: Patients who received neoadjuvant therapy before breast surgery in our hospital, patients who received SLNB without ALND or directly received ALND during breast surgery, the pathological type was ductal carcinoma in situ, a distant metastasis, and male breast cancer patient.

**Fig. 1:**
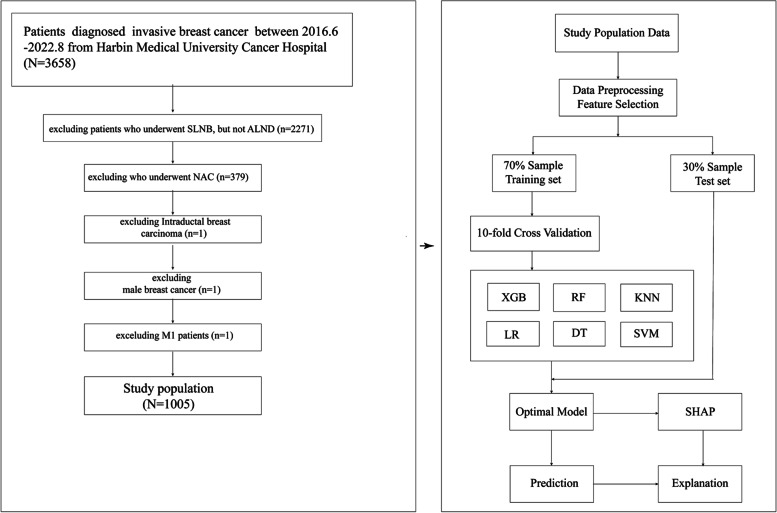
The flow chart of patients selection and the flow chart for the development, evaluation, and explanation of models

Finally, a total of 1005 breast cancer patients were included. Their details are shown in Fig. 1.

### Axillary lymph node status management

Methylene blue dye was injected into the intradermal, subcutaneous, areolar, and glandular areas (peritumor, intratumor, subtumor) 10–15 min before breast cancer surgery (Jichuan Pharmaceutical Group, China), or a carbon nanosuspension was injected into the subserous membrane along the peritumoral site at 4–6 points (Chongqing Lemei Pharmaceutical, China) during surgery to facilitate the localization of SLNB. Sentinel and nonsentinel lymph nodes were evaluated in hematoxylin-eosin (HE)-stained sections that were fixed with 10% formalin and embedded in paraffin. After fixation, successive sections of the lymph nodes were obtained for definitive analysis of lymph node status.

### Classification

An estrogen receptor (ER) immunohistochemical (IHC) detection degree of < 1% nuclear staining was interpreted as ER negativity, and an IHC-positive degree between 1 and 10% nuclear staining was interpreted as ER weak positivity. An IHC-positive degree of > 10% nuclear staining was interpreted as ER positivity [21]. Progesterone receptor (PR) was negative if its IHC-positive degree was < 1%, weakly positive if its IHC-positive degree was between 1 and 20%, and positive if its IHC-positive degree was > 20% [22]. A human epidermal growth factor receptor-2 (HER-2) IHC result of 0 was defined as HER-2 negative. Low HER-2 expression was defined as a HER-2 IHC result of 1+ or a HER-2 IHC result of 2+ along with negative fluorescence in situ hybridization (FISH). A HER-2 IHC result of 3+ or a HER-2 IHC result of 2+ with positive FISH was defined as HER-2 positivity [23]. The Ki-67 expression level was divided into the Ki-67 ≤ 14% group and the Ki-67 > 14% group [24]. According to the results of IHC, all patients were divided into luminal A, luminal B, triple-negative breast cancer (TNBC), and HER-2 overexpression groups [22].

According to current American Joint Committee on Cancer (AJCC) standards [25], single tumor cells or maximum tumor diameter < 2 mm in axillary lymph nodes was defined as node negative, and tumor diameter ≥ 2 mm was defined as node positive. Pathological lymph node staging (pN) was determined according to the number of positive axillary lymph nodes. The staging was as follows: pN0 meant no axillary lymph node metastasis, pN1 meant 1–3 axillary lymph node metastases, pN2 meant 4–9 axillary lymph node metastases, and pN3 meant more than 9 axillary lymph node metastases.

Since the patients included in this study were Chinese women with breast cancer, body mass index (BMI) was classified into different groups according to the standards of the Chinese Health Commission. BMI = weight (kg)/height (m^2^), and the underweight group was defined as BMI < 18.5 kg/m^2^. BMI between 18.5 and 23.9 kg/m^2^ was the normal group, BMI between 24 and 27.9 kg/m^2^ was the overweight group, and BMI ≥ 28 kg/m^2^ was the obesity group.

Considering the small number of patients with other types of breast cancer included, the patients were divided into infiltrating ductal carcinoma and other types of carcinoma according to pathological type, including invasive lobular carcinoma (18 patients), invasive micropapillary carcinoma (3 patients), ductal carcinoma in situ with microinvasion (3 patients), and mucinous carcinoma (2 patients).

### Data preprocessing and feature selection

The k-nearest neighbor imputer (KNNImputer) was used to supplement parameters with missing values less than 30% [26]. Recursive feature elimination was applied to select the best variables (Fig. S1). The best number of variables was 12: age, BMI, pregnancy history, nipple retraction, single/multiple tumors, cT stage, blood flow signal of tumor, cN stage, ultrasound (US) BI-RADS classification, mammography (MG) BI-RADS classification, SLN group, and SLN-positive ratio.

### Model development

This study introduced six ML algorithms, including extreme gradient boosting (XGBoost), logistic regression (LR), support vector machine (SVM), k-nearest neighbor (KNN), random forest (RF), and decision tree (DT).

The LR model is usually applied to explore how characteristics influence binary variables [27]. In the face of a regression or classification problem, the cost function is established, the optimal model parameters are iteratively solved by the optimization method, and then the quality of the resulting model is verified by testing.

SVM is applied to classify things with multidimensional attributes into two categories [28]. It is a supervised learning model that is commonly used for pattern recognition, classification, and regression analysis. Based on structural risk minimization theory, it constructs the optimal hyperplane in the feature space so that the learner is globally optimized, and the expectation of the whole sample space satisfies a certain upper bound with a certain probability.

KNN is one of the most commonly used nonparametric classification techniques. Its working premise is that if most of the nearest k samples to a given sample belong to a certain class in the feature space, then they all must belong to the same class. The KNN method is only related to a very small number of adjacent samples in the class decision. Because the KNN method mainly depends on a few neighboring samples, rather than the method of discriminating the class domain to determine the category, the KNN method is more suitable for dividing the sample with more crossover or overlap of class domains [29].

Classifiers that use multiple trees to train and predict samples are called RF classifiers, which reduces training variance and improves integration and generalization capabilities [30]. Its training can be highly parallelized, which has advantages for large-sample training speed in the era of big data. Since the decision tree nodes that divide the features can be randomly selected, the model can still be trained efficiently even when the sample feature dimension is very high.

The DT algorithm can be divided layer by layer according to the characteristics of the data until all the characteristics are divided, so it can be used to solve classification and regression problems [31]. It is a kind of nonparametric supervised learning that is easy to understand, applicable to all kinds of data, and has good performance in solving various problems, especially various integrated algorithms with tree models as the core. It is widely used in various industries and fields.

XGBoost is an ML technique that can process missing data and build accurate prediction models from weak prediction models [32]. It is good at capturing dependencies between complex data, can obtain effective models from large-scale datasets, and supports multiple systems and languages in practical terms.

### Statistical methods

All patients were randomly divided into training and testing sets at a 7:3 ratio (Fig. 1). The ML prediction model was developed in the training set and optimized by using 10-fold cross-validation. The AUC, accuracy, recall rate, F1 value, and precision were used to evaluate the ability of each ML model. Brier scores were applied to evaluate the overall performance of the model [33]. Pearson’s *χ*^2^ or Fisher’s exact test was used for intergroup analysis. Univariate and multivariate analyses were performed using logistic regression. Based on multivariable logistic regression analysis, a nomogram was built, whose accuracy was determined by calculating its C-index. The internal verification was carried out by the bootstrap method, and the difference between the actual value and the predicted value obtained from the column chart was analyzed graphically. To more intuitively explain the optimal ML model, we introduce the SHAP framework, whose interpretability has been demonstrated in many cancers [18, 34–36]. It can demonstrate the contributions of various variables in any ML model to the outcome event [20]. All statistics were performed using Python 3.9 and R language 4.1.2. *P*< 0.05 was considered statistically significant.

## Results

### Clinicopathologic features of patients

A total of 1005 breast cancer patients with a median age of 51 years were enrolled in this study, of whom 829 (82.5%) underwent mastectomy and 176 (17.5%) underwent breast-conserving surgery. NSLNM occurred in 338 cases and not in 667 cases (Table 1). Ninety-nine patients (9.9%) were classified as luminal A, 799 patients (79.5%) were classified as luminal B, 47 patients (4.7%) were classified as TNBC, and 60 patients (6.0%) were classified as HER-2 overexpressing. Most of the included patients were patients with stages T1 and T2 (cT1 and cT2), with a total of 466 patients (46.4%) at cT1 and 512 patients (50.9%) at cT2. Most cancers were pN1 (562 cases (55.9%)). There were 340 cases (33.8%) in the Ki-67 ≤ 14 group and 665 cases (66.2%) in the Ki-67 ≥ 14 group. There were 609 cases (60.6%) without lymphatic vascular infiltration (LVI) and 396 cases (39.4%) with LVI. There were 789 (78.5%) patients with one or two sentinel lymph node metastases (SLNMs) and 113 (11.2%) patients with three or more SLNBs. Notably, 103 patients (10.2%) did not develop SLNM, but 21 of them (20.4%) did develop NSLNM. There were 738 patients (73.4%) with a sentinel node-positive ratio (the number of positive SLNs to the total SLN ratio) ≤ 0.5 and 267 cases (26.6%) with an SLN-positive ratio > 0.5. BMI, cT, cN, ultrasonic (US) BI-RADS classification, mammography (MG) BI-RADS classification, pT, pN, SLN status, number of SLNMs, sentinel lymph node-positive ratio, and LVI were correlated with NSLNM (*p* < 0.05, Table 1).

**Table 1:** The relationship between characteristics and non-SLN metastasis

Characteristics	Overall	Without non-SLN metastasis	With non-SLN metastasis	*p*
	*N* = 1005	*n* = 667	*n* = 338	
**Age (median [IQR])**	51.00 (45.00, 59.00)	51.00 (45.00, 60.00)	51.00 (46.00, 59.00)	0.951
**Position (%)**				0.592
Left	498 (49.6)	326 (48.9)	172 (50.9)	
Right	507 (50.4)	341 (51.1)	166 (49.1)	
**BMI (%)**				**0.004**
< 18.5	23 (2.3)	13 (1.9)	10 (3.0)	
18.5–23.9	465 (46.3)	332 (49.8)	133 (39.3)	
24–27.9	385 (38.3)	248 (37.2)	137 (40.5)	
≥ 28	132 (13.1)	74 (11.1)	58 (17.2)	
**Pregnant (%)**				0.901
0	48 (4.8)	33 (4.9)	15 (4.4)	
1	652 (64.9)	430 (64.5)	222 (65.7)	
≥ 2	305 (30.3)	204 (30.6)	101 (29.9)	
**Menopause (%)**				0.828
No	488 (48.6)	326 (48.9)	162 (47.9)	
Yes	517 (51.4)	341 (51.1)	176 (52.1)	
**Nipple retraction (%)**				0.104
No	954 (94.9)	639 (95.8)	315 (93.2)	
Yes	51 (5.1)	28 (4.2)	23 (6.8)	
**Nipple discharge (%)**				1
No	972 (96.7)	645 (96.7)	327 (96.7)	
Yes	33 (3.3)	22 (3.3)	11 (3.3)	
**Number of tumor (%)**				0.487
Single focal	900 (89.6)	601 (90.1)	299 (88.5)	
Multi-focal	105 (10.4)	66 (9.9)	39 (11.5)	
**cT (%)**				**0.042**
1	466 (46.4)	328 (49.2)	138 (40.8)	
2	512 (50.9)	321 (48.1)	191 (56.5)	
3	19 (1.9)	14 (2.1)	5 (1.5)	
4	8 (0.8)	4 (0.6)	4 (1.2)	
**Aspect ratio (%)**				0.867
< 1	924 (91.9)	615 (92.2)	309 (91.4)	
> 1	63 (6.3)	41 (6.1)	22 (6.5)	
= 1	18 (1.8)	11 (1.6)	7 (2.1)	
**US tumor borderline (%)**				0.200
Clear	101 (10.0)	75 (11.2)	26 (7.7)	
Lack of clarity	246 (24.5)	163 (24.4)	83 (24.6)	
Blurring	658 (65.5)	429 (64.3)	229 (67.8)	
**US tumor form (%)**				0.080
Rule	57 (5.7)	42 (6.3)	15 (4.4)	
Underrule	54 (5.4)	42 (6.3)	12 (3.6)	
Irregularity	894 (89.0)	583 (87.4)	311 (92.0)	
**US tumor blood (%)**				0.164
No	183 (18.2)	130 (19.5)	53 (15.7)	
Yes	822 (81.8)	537 (80.5)	285 (84.3)	
**cN (%)**				**0.001**
0	371 (36.9)	272 (40.8)	99 (29.3)	
1	562 (55.9)	348 (52.2)	214 (63.3)	
2	3 (0.3)	3 (0.4)	0 (0.0)	
3	69 (6.9)	44 (6.6)	25 (7.4)	
**US BI-RADS (%)**				**< 0.001**
3	24 (2.4)	21 (3.1)	3 (0.9)	
4	811 (80.7)	554 (83.1)	257 (76.0)	
5	163 (16.2)	87 (13.0)	76 (22.5)	
6	7 (0.7)	5 (0.7)	2 (0.6)	
**MG calcification (%)**				0.653
No	257 (25.6)	174 (26.1)	83 (24.6)	
Yes	748 (74.4)	493 (73.9)	255 (75.4)	
**MG BI-RADS (%)**				**0.016**
3	97 (9.7)	75 (11.2)	22 (6.5)	
4	741 (73.7)	485 (72.7)	256 (75.7)	
5	133 (13.2)	80 (12.0)	53 (15.7)	
6	34 (3.4)	27 (4.0)	7 (2.1)	
**pT (%)**				**0.026**
1	629 (62.6)	433 (64.9)	196 (58.0)	
2	361 (35.9)	228 (34.2)	133 (39.3)	
3	7 (0.7)	2 (0.3)	5 (1.5)	
4	8 (0.8)	4 (0.6)	4 (1.2)	
**pN (%)**				**< 0.001**
0	82 (8.2)	82 (12.3)	0 (0.0)	
1	734 (73.0)	577 (86.5)	157 (46.4)	
2	130 (12.9)	8 (1.2)	122 (36.1)	
3	59 (5.9)	0 (0.0)	59 (17.5)	
**SLNs group (%)**				**0.004**
Negative	103 (10.2)	82 (12.3)	21 (6.2)	
Positive	902 (89.8)	585 (87.7)	317 (93.8)	
**SLNM (%)**				**< 0.001**
0	103 (10.2)	82 (12.3)	21 (6.2)	
1/2	789 (78.5)	549 (82.3)	240 (71.0)	
≥ 3	113 (11.2)	36 (5.4)	77 (22.8)	
**Ratio of no. of positive SLNs to total no. of SLNs (%)**				**< 0.001**
≤ 0.5	738 (73.4)	561 (84.1)	177 (52.4)	
> 0.5	267 (26.6)	106 (15.9)	161 (47.6)	
**Grade (%)**				0.114
1	18 (1.8)	16 (2.4)	2 (0.6)	
2	768 (76.4)	504 (75.6)	264 (78.1)	
3	219 (21.8)	147 (22.0)	72 (21.3)	
**Pathological type (%)**				0.345
Invasive ductal carcinoma	979 (97.4)	647 (97.0)	332 (98.2)	
Others	26 (2.6)	20 (3.0)	6 (1.8)	
**Subtype (%)**				0.650
Luminal A	99 (9.9)	68 (10.2)	31 (9.2)	
Luminal B	799 (79.5)	523 (78.4)	276 (81.7)	
TNBC	47 (4.7)	34 (5.1)	13 (3.8)	
HER-2 overexpression	60 (6.0)	42 (6.3)	18 (5.3)	
**ER (%)**				0.118
Negative	153 (15.2)	108 (16.2)	45 (13.3)	
Low	19 (1.9)	16 (2.4)	3 (0.9)	
High	833 (82.9)	543 (81.4)	290 (85.8)	
**PR (%)**				0.105
Negative	211 (21.0)	150 (22.5)	61 (18.0)	
Low	138 (13.7)	83 (12.4)	55 (16.3)	
High	656 (65.3)	434 (65.1)	222 (65.7)	
**HER2 (%)**				0.610
Negative	307 (30.5)	197 (29.5)	110 (32.5)	
Low	539 (53.6)	362 (54.3)	177 (52.4)	
Positive	159 (15.8)	108 (16.2)	51 (15.1)	
**Ki-67 (%)**				0.051
≤ 14	340 (33.8)	240 (36.0)	100 (29.6)	
> 14	665 (66.2)	427 (64.0)	238 (70.4)	
**P53 (%)**				0.106
Negative	351 (34.9)	245 (36.7)	106 (31.4)	
Positive	654 (65.1)	422 (63.3)	232 (68.6)	
**LVI (%)**				**< 0.001**
No	609 (60.6)	444 (66.6)	165 (48.8)	
Yes	396 (39.4)	223 (33.4)	173 (51.2)	
**Surgical method (%)**				0.818
BCS	176 (17.5)	115 (17.2)	61 (18.0)	
Mastectomy	829 (82.5)	552 (82.8)	277 (82.0)	

*Abbreviations*: *BMI* Body mass index, *US* Ultrasound, *MG* Mammography, *SLN* Sentinel lymph node, *SLNM* Sentinel lymph node metastasis, *ER* Estrogen receptor, *PR* Progesterone receptor, *HER2* Human epidermal growth factor receptor 2, *LVI* Lymphatic vascular infiltration, *BCS* Breast-conserving surgery

### Univariate and multivariate logistic regression analysis in the training set

A total of 1005 patients were randomly classified into the training set (703 patients) and the test set (302 patients) in a ratio of 7:3. In the training set, univariate analysis showed that cN1/cN2/cN3 was more prone to NSLNM than cN0 (OR = 1.5, 95% CI: 1.07–2.09, *p* = 0.018). NSLNM was more likely to occur in MG BI-RADS 4 than in MG BI-RADS 3 (OR = 1.77, 95% CI: 1.02–3.05, *p* = 0.041). Compared with patients with negative SLNs, patients with positive SLNs were more likely to develop NSLNM (OR = 2.38, 95% CI: 1.3–4.36, *p* = 0.005). Compared with patients with an SLN-positive ratio ≤ 0.5, patients with an SLN-positive ratio > 0.5 were more likely to develop NSLNM (OR = 3.82, 95% CI: 2.69–5.43, *p* < 0.001). A variance inflation factor (VIF) < 10 indicates that there is no multicollinearity among different parameters [37]. Parameters with *p* < 0.05 from the univariate analysis were included in the multivariate analysis, and the results showed that the SLN-positive rate was an independent predictor of NSLNM (OR = 3.51, 95% CI: 2.43–5.05, *p* < 0.001) (Table 2).

**Table 2:** Relationship between training set characteristics and NSLNM

**Characteristics**	**Univariate**		**Multivariate**	
	**OR (95% *CI*)**	***P***	**OR (95% *CI*)**	***P***
**Age (median [IQR])**	1 (0.98–1.02)	0.935		
**Position (%)**				
Left	1			
Right	1.03 (0.75–1.41)	0.856		
**BMI (%)**				
< 18.5	1			
18.5–23.9	0.59 (0.22–1.61)	0.306		
24–27.9	0.77 (0.28–2.11)	0.608		
≥ 28	0.95 (0.34–2.62)	0.917		
**Pregnant (%)**				
0	1			
1	1.02 (0.48–2.17)	0.961		
≥ 2	0.85 (0.39–1.87)	0.691		
**Menopause (%)**				
No	1			
Yes	1.1 (0.81–1.51)	0.542		
**Nipple retraction (%)**				
No	1			
Yes	1.41 (0.7–2.84)	0.338		
**Nipple discharge (%)**				
No	1			
Yes	0.45 (0.13–1.59)	0.216		
**Number of tumor (%)**				
Single focal	1			
Multi-focal	1.51 (0.92–2.49)	0.104		
**cT (%)**				
1	1			
2	1.31 (0.95–1.81)	0.095		
3	1.04 (0.35–3.07)	0.942		
4	2.29 (0.32–16.49)	0.411		
**Aspect ratio (%)**				
< 1	1			
> 1	1.35 (0.7–2.59)	0.369		
= 1	1.26 (0.41–3.91)	0.684		
**US tumor borderline (%)**				
Clear	1			
Lack of clarity	1.46 (0.79–2.68)	0.228		
Blurring	1.46 (0.84–2.56)	0.181		
**US tumor form (%)**				
Rule	1			
Underrule	0.82 (0.3–2.26)	0.699		
Irregularity	1.45 (0.71–2.94)	0.308		
**US tumor blood (%)**				
No	1			
Yes	1.03 (0.69–1.54)	0.895		
**cN**				
cN0	1			
cN1/cN2/cN3	1.5 (1.07–2.09)	**0.018**	1.22 (0.86–1.74)	0.269
**US BI-RADS (%)**				
3	1			
4	2.02 (0.57–7.18)	0.277		
5	3.4 (0.92–12.57)	0.067		
6	2.89 (0.32–25.7)	0.341		
**MG calcification (%)**				
No	1			
Yes	1.15 (0.8–1.65)	0.453		
**MG BI-RADS (%)**				
3	1			
4	1.77 (1.02–3.05)	**0.041**	1.68 (0.95–2.97)	0.077
5	1.88 (0.97–3.66)	0.063	1.92 (0.96–3.86)	0.067
6	1.03 (0.36–2.95)	0.955	0.9 (0.3-2.7)	0.849
**SLNs group (%)**				
Negative	1			
Positive	2.38 (1.3–4.36)	**0.005**	1.64 (0.88–3.05)	0.119
**Ratio of no. of positive SLNs to total no. of SLNs (%)**				
≤ 0.5	1			
> 0.5	3.82 (2.69–5.43)	**< 0.001**	3.51 (2.43–5.05)	**< 0.001**

*Abbreviations*: *BMI* Body mass index, *US* Ultrasound, *MG* Mammography, *SLN* Sentinel lymph node

### Machine learning model construction and performance comparison

**Fig. 2:**
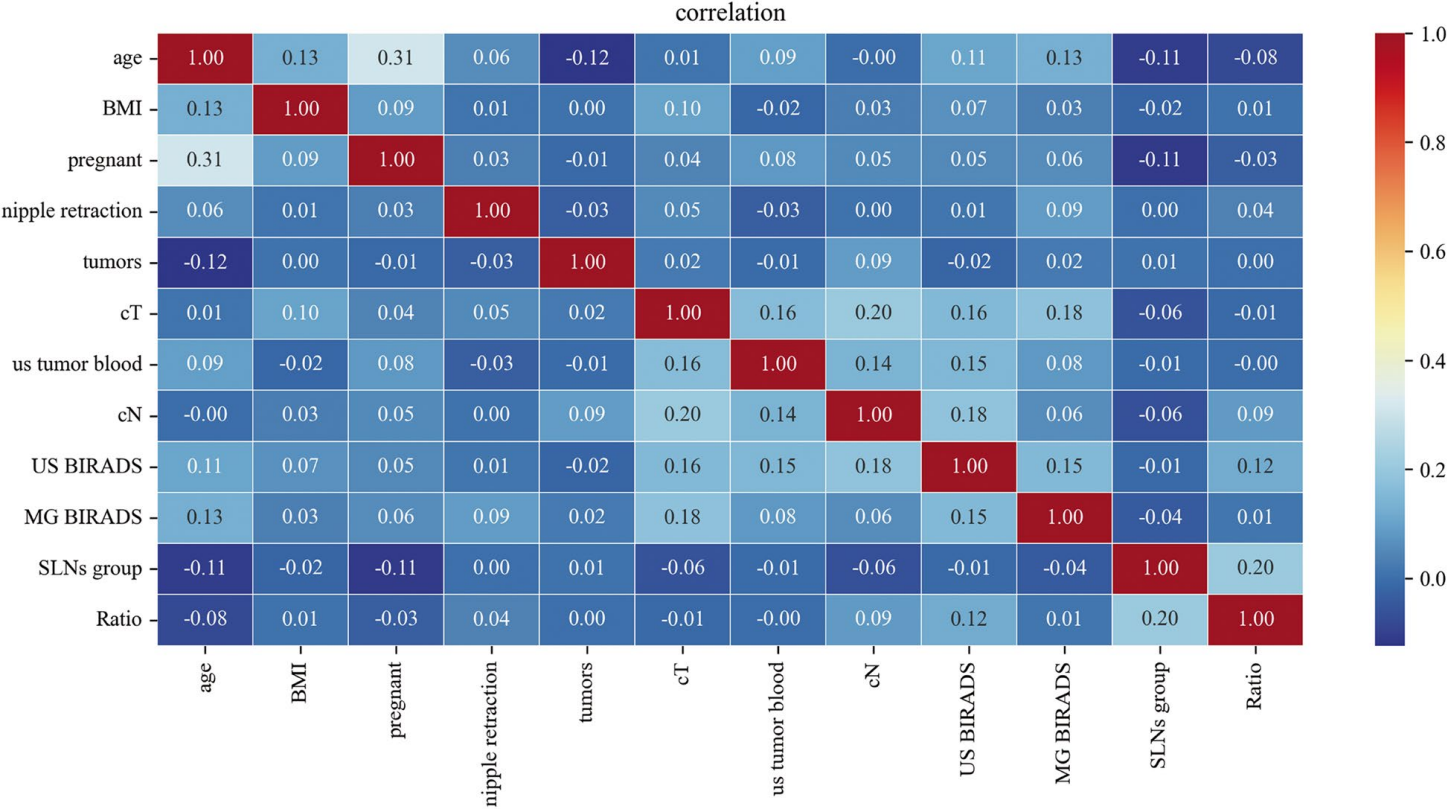
The relationship between different variables

**Fig. 3:**
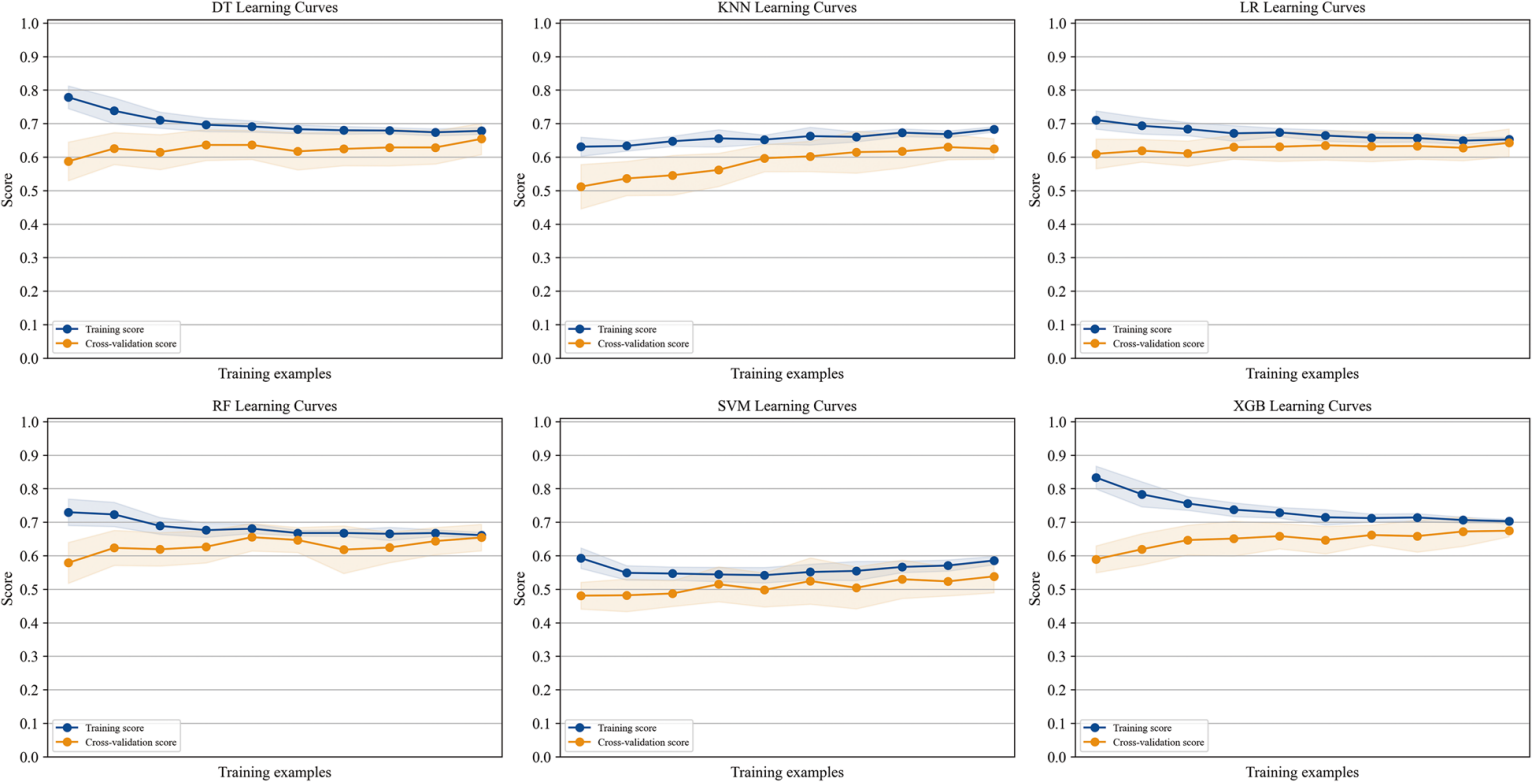
Learning curves of different machine learning models

**Fig. 4:**
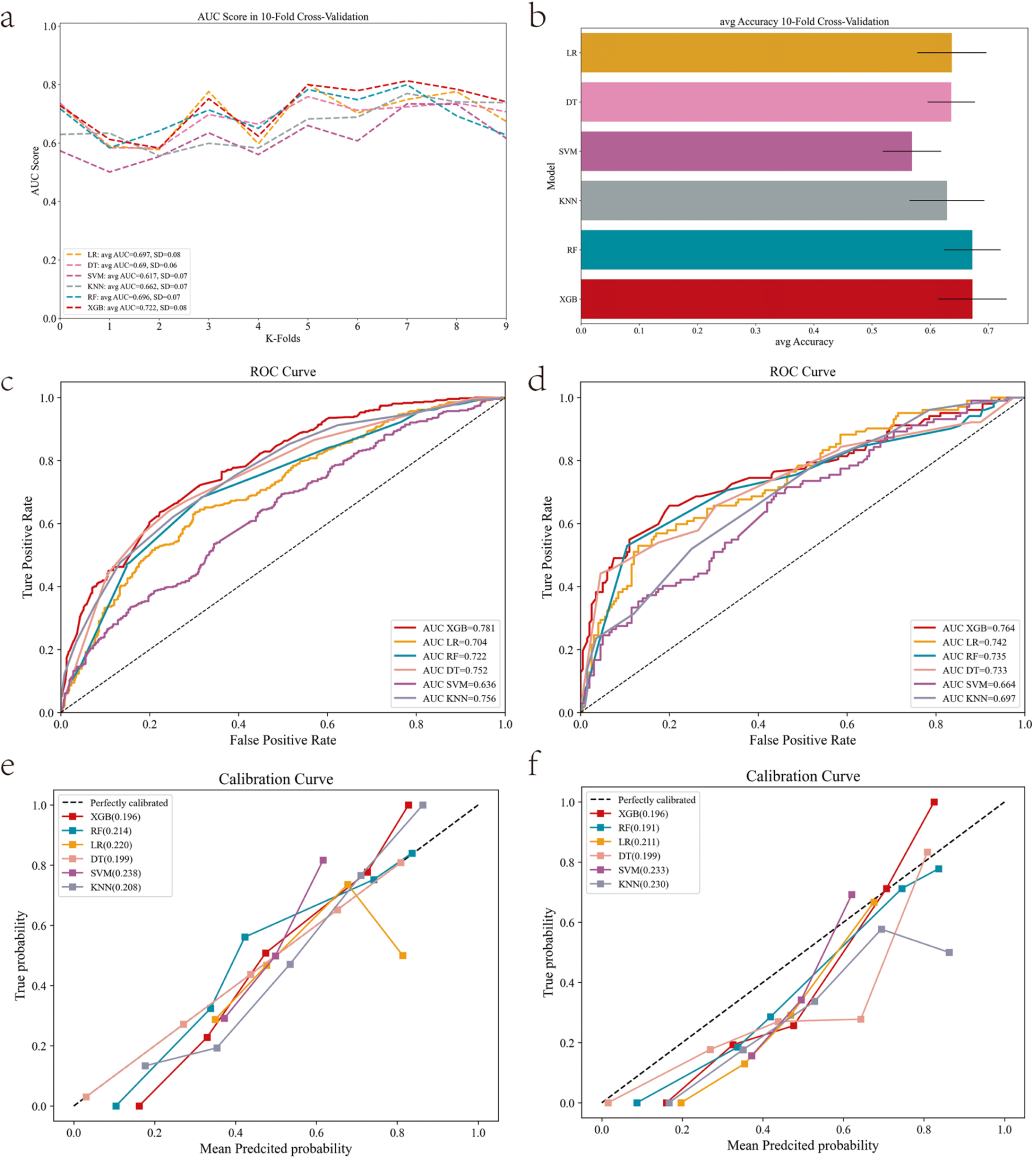
Performance comparison of different machine learning models

**Fig. 5:**
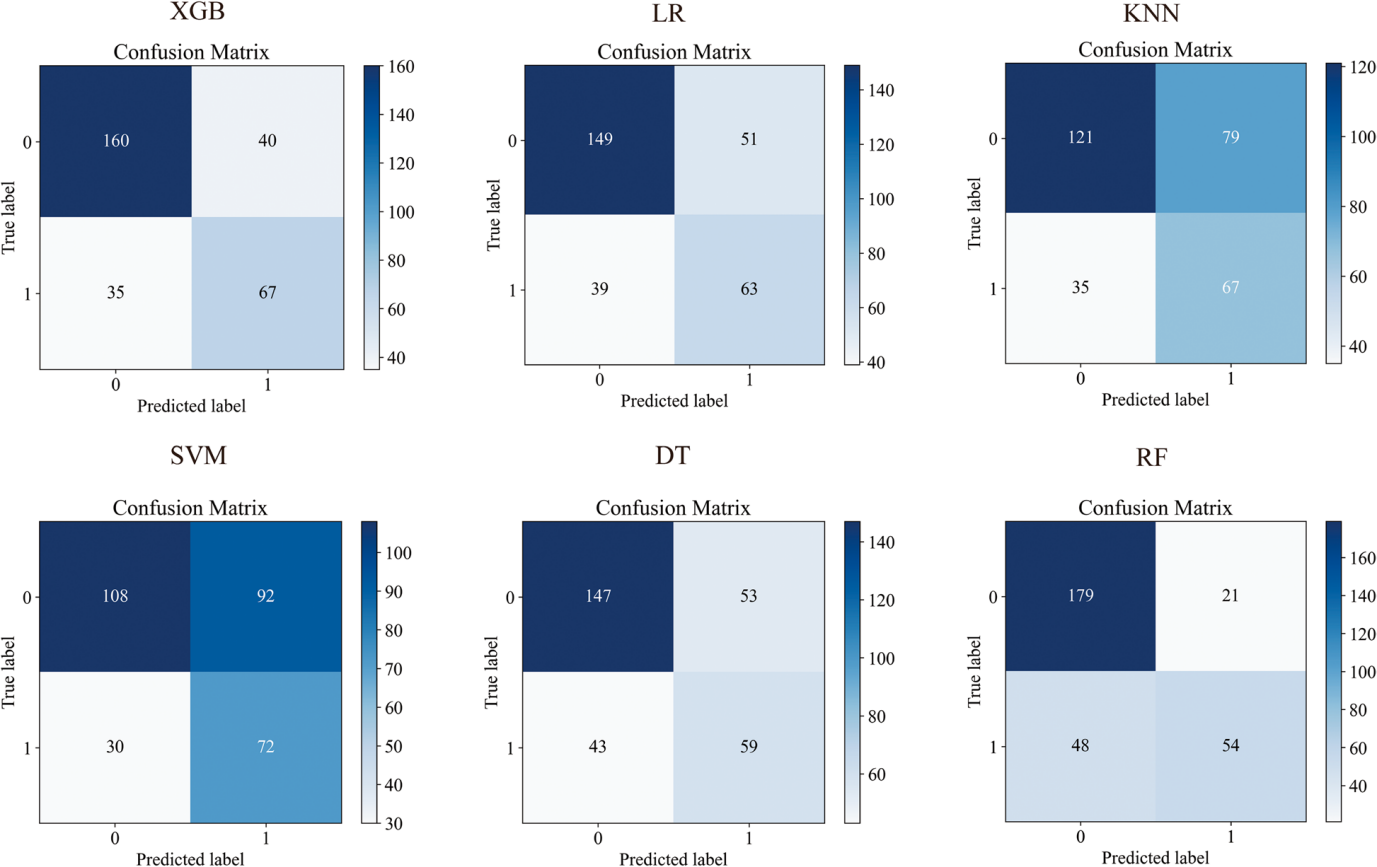
The confusion matrix of different ML models

Twelve variables were selected to develop ML models. The relationships between different variables are shown in Fig. 2. Based on the above 12 variables, six ML models were developed on the training set, and learning curves showed that there was no overfitting of these six machine learning models (Fig. 3). Therefore, we further compared the performance of different ML models using the AUC value, accuracy, precision, F1 value, and Brier score. The results show that in the training set with 10-fold cross-validation, the average AUC value of the XGBoost model was the largest (0.722, Fig. 4a), and its accuracy was the highest (0.673, Fig. 4b). Moreover, in both the training set and the test set, the AUC value of the XGBoost model was the largest, at 0.781 (Fig. 4c) and 0.764 (Fig. 4d), respectively. The Brier score was the smallest in the training set and the second smallest in the test set, at 0.196 (Fig. 4e) and 0.191 (Fig. 4f). In the test set, the accuracy and precision of the XGBoost model were the second largest, at 0.752 and 0.723, respectively. The recall rate and F1 value of the XGBoost model were the highest, at 0.728 and 0.726, respectively (Table 3). The positive predictive value and negative predictive value of the XGBoost model were highly consistent with the real values (Fig. 5). In conclusion, of the six ML models tested, the XGBoost model demonstrated the best performance.

**Table 3:** Results of NSLNM predicted by different ML

**Indicators**	**LR**	**DT**	**SVM**	**KNN**	**RF**	**XGB**
**Accuracy**	0.702	0.682	0.596	0.623	0.772	0.752
**Precision**	0.673	0.650	0.611	0.617	0.754	0.723
**Recall**	0.681	0.657	0.623	0.631	0.712	0.728
**F1 score**	0.676	0.653	0.590	0.610	0.724	0.726

*Abbreviations*: *LR* Logistic regression model, *DT* Decision tree model, *SVM* Support vector machine model, *KNN* K-nearest neighbor model, *RF* Random forest model, *XGB* Extreme gradient boosting model

Establishment of the nomogram and performance comparison with the XGBoost model

Based on multivariate logistic regression analysis, the SLN-positive ratio was an independent predictor of NSLNM. In a previous study, SLN status was also correlated with NSLNM [38]. Therefore, these two variables were applied to develop the nomogram. The C-index of the nomogram in the training set and test set was 0.706 and 0.647, respectively. After internal verification by the bootstrap method, the C-index in the training set and test set was similar, at 0.706 and 0.646, respectively. Figure S2a shows a nomogram for predicting NSLNM based on the SLN-positive ratio and SLN group. Based on the scores from the different states of the nomogram’s variables, the probability of NSLNM for a certain patient can be obtained. The AUC values of this model in the training set and the test set were 0.647 (Fig. S2b) and 0.706 (Fig. S2c), respectively. The deviation between the predicted value and the actual value in the training set and the test set was somewhat large (Fig. S2d, e). In the training and test sets, the AUC value of XGBoost was larger than that of the nomogram (0.781 vs. 0.647; 0.764 vs. 0.706; Table S1). These results showed that the XGBoost model was superior to the nomogram in predicting NSLNM.

### Interpretability of the XGBoost model

**Fig. 6:**
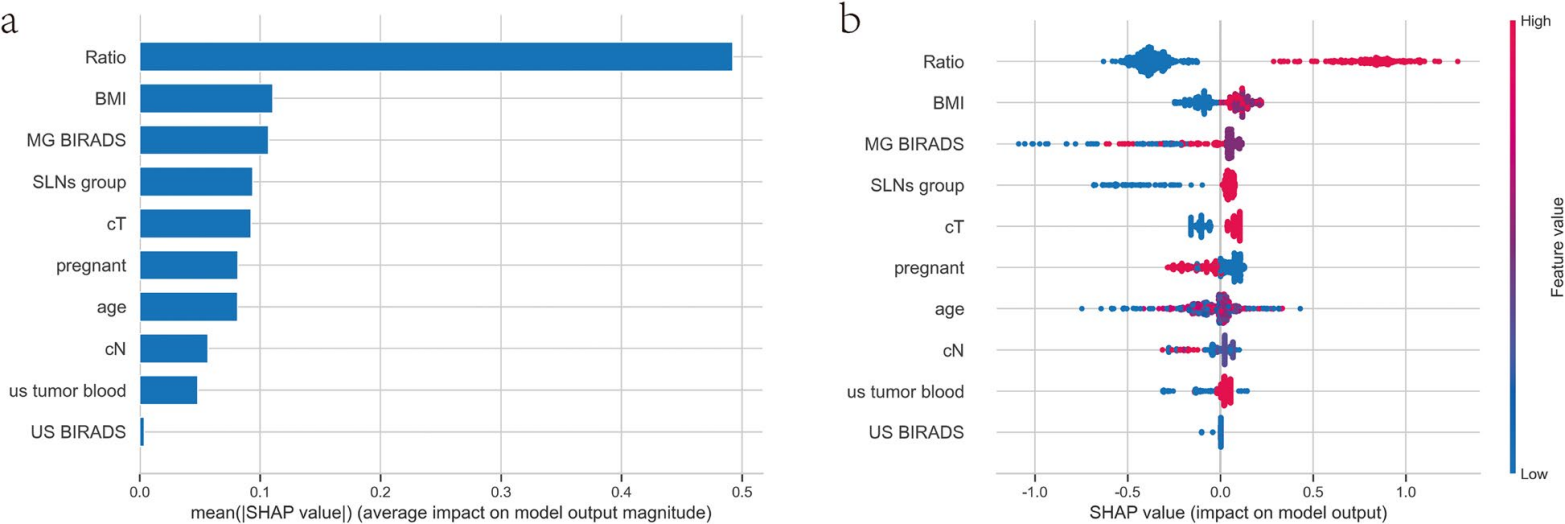
SHAP summary of XGBoost model

Based on the above results, XGBoost was the best model to predict NSLNM. To make this model and its prediction easier to understand, this study makes use of the SHAP framework. Figure 6a shows the first ten characteristic parameters affecting NSLNM: SLN-positive ratio, BMI, MG BI-RADS classification, SLN group, cT, number of births, age, cN, US blood flow signal of tumor, and US BI-RADS classification. To explore how these characteristics affect NSLNM, SHAP values are further used for interpretation (Fig. 6b). The SHAP value (*X*-axis) represents the degree to which the feature influenced NSLNM, and the feature ranking (Y-axis) represents the size of the feature values. Red dots represent higher values, and blue dots represent lower values. The results show that compared with an SLN-positive ratio ≤ 0.5, a sentinel node-positive ratio > 0.5 was more likely to be found along with NSLNM. Compared with the low-BMI group and the normal-BMI group, the overweight and obesity group was more likely to develop NSLNM. Compared with the lower class of MG BI-RADS, the higher class of MG BI-RADS was more likely to be found with NSLNM. NSLNM was more likely to occur in the SLN-positive group than in the SLN-negative group. NSLNM was more likely to occur with higher clinical T stage than with lower clinical T stage.

**Fig. 7:**
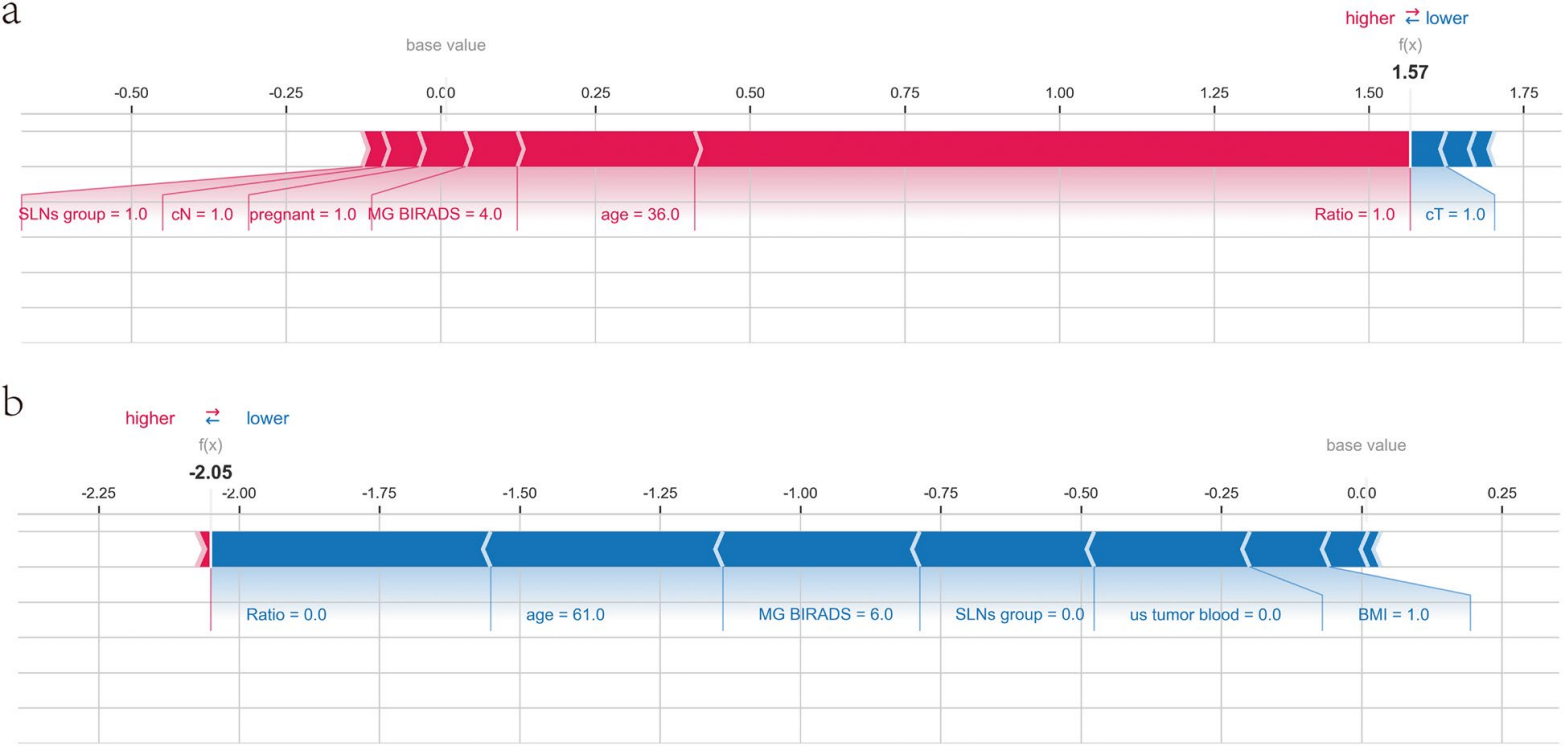
NSLNM prediction of two typical patients

This study also individualized the interpretation of the model and took two typical examples to verify the accuracy of XGBoost: one patient with actual NSLNM (Fig. 7a) and one patient without NSLNM (Fig. 7b). Arrows demonstrate the effects of different variables on the outcome prediction. Red and blue arrows show whether the variable was likely to occur (red) or not (blue). The combined effects of all variables provided the final SHAP value, corresponding to the predicted score. The patient with NSLNM had a high SHAP value of 1.57 and a high prediction score of 0.83. The patient without NSLNM had a low SHAP value of −2.05 and a low prediction score of 0.11.

## Discussion

In this study, we constructed six ML models to predict NSLNM using preoperative and intraoperative clinicopathological features and compared their performance. The XGBoost model showed the best performance, and its predictive ability was also superior to that of the nomogram. The XGBoost model was well explained through the SHAP framework.

In previous studies, LVI, grade, pathological tumor size, and molecular typing of breast cancer were often included in prediction model to predict NSLNM [39–42]. Although the inclusion of these postoperative parameters improved the prediction accuracy, the difficulty in obtaining these parameters preoperatively and intraoperatively may limit their clinical application. A previous study used clinical tumor size to establish predictive models [43]. Therefore, clinical tumor size was put into the predictive models in this study. Murata, T et al. included 804 patients with operable primary breast cancer and showed that NSLNM was more likely to occur with an SLN-positive ratio of ≥ 0.5 than with an SLN-positive ratio of < 0.5 (*p* = 0.024) [44]. Wang Nana et al. retrospectively analyzed 495 patients and found that patients in the SLN-positive group were more likely to develop NSLNM than those in the SLN-negative group (*p* < 0.001) [41]. This study also demonstrated that the SLN-positive rate was an independent predictor of NSLNM.

Some scholars [45] have found that NSLNM was closely related to the ultrasound tumor boundary and blood flow signal (*p* = 0.038, *p* = 0.036). This study had similar results, 26 patients (7.7%) with clear ultrasound tumor boundaries had NSLNM, while 83 patients (24.6%) with ambiguous ultrasound tumor boundaries and 229 patients (67.8%) with unclear ultrasound tumor boundaries had NSLNM. Patients with ambiguous or unclear tumor boundaries were more likely to develop NSLNM. In the patients with NSLNM, most patients (84.3%) showed a blood flow signal. The above parameters were not independent predictors of NSLNM, which may be attributed to the fact that SLN-negative patients were also included in this study.

Kuo YL et al. retrospectively analyzed 1496 malignant breast cancer patients and established a nomogram to predict NSLNM. The model showed good predictive performance, and the AUC value of the model was 0.738 [46], but it is not clear whether it was better than the ML model. Mi DU et al. developed an ML model to predict 3-year and 5-year disease-specific survival for oral and pharyngeal cancers and compared its performance with conventional Cox regression, showing that the ML model had better predictive performance [47]. However, no such comparison has been made in breast cancer for predicting NSLNM. In this study, for the first time, the prediction performance of NSLNM was compared between the ML model and nomogram. The results demonstrated that the AUC value of the XGBoost model was larger than that of the traditional nomogram (0.781 vs. 0.647; 0.764 vs. 0.706). Compared with traditional regression models, ML models can more accurately identify and analyze the potential relationships between different variables, and their predictive accuracy is particularly suitable for achieving individualized therapy and predictive medicine [48], which will help us better solve clinical problems.

A deep learning radiomics model has been developed to predict the risk of NSLNM. Its sensitivity for NSLNM was 98.4% (95% CI: 95.6–99.9%), and its negative predictive value was 91.7% (95% CI: 88.8–97.9%) in the validation set [17]. This model has good predictive ability, but the lack of explanation of the model makes it impossible for readers to intuitively understand the prediction results of the ML model, and its complex region of interest (ROI) drawing also limits its application for clinical breast surgeons. In this study, six powerful ML models were developed using clinicopathological features that are easy to obtain, and their predictive performance for NSLNM was compared. All models showed good predictive performance; the XGBoost model is the best. We visualized the optimal model with SHAP values and graphs. The summary charts show the effects of different variables on NSLNM, among which the SLN-positive ratio had the greatest impact on NSLNM. Compared with an SLN-positive ratio ≤ 0.5, an SLN-positive ratio > 0.5 was more likely to produce NSLNM. Two typical patients (one with NSLNM and one without NSLNM) were also explained using force diagrams.

Some studies [11–13] showed that 40–60% of breast cancer patients with SLNB further underwent ALND, even if no other lymph node metastasis was found. Some breast cancer patients chose to directly undergo ALND due to poor finances condition. With ALND comes the problem of lymphedema, which limits the upper limb function of breast cancer patients, leading to worse working ability and lower income, creating a vicious cycle. On the other hand, some patients were found to have NSLNM with negative SLNs (6.2% in this study), which could lead to a second surgery of the axilla. Therefore, accurate prediction of NSLNM is necessary. The XGBoost model in this study showed powerful predictive ability, which could help us avoid overtreatment or undertreatment. There is still a long way to go before this model can be applied to real-world medical settings because it still needs to be tested in different populations. In addition, developing a software application (APP) based on this model will be a difficult and time-consuming project.

Although the XGBoost model developed here can well predict NSLNM, this study has some limitations. First, this is a retrospective study conducted at a single institution. The inclusion of multicenter data would be more conducive to model validation. Second, with the exception of the group with breast invasive ductal carcinoma, few patients had other pathological types of breast cancer. If the sample size of patients with other pathological types of breast cancer can be increased, the probability of occurrence of NSLNM in different pathological types can be better compared, and the ML model developed will be more suitable for clinical practice.

## Conclusion

The optimal ML model XGBoost was developed using preoperative and intraoperative clinicopathological features and was superior to the traditional nomogram in predicting NSLNM. The SHAP framework can explain how the best model works, intuitively display the influence of characteristic variables on NSLNM, realize the clinical translation of machine learning technology, and assist clinicians in making more individualized and accurate diagnosis and treatment plans.

### Supplementary Information

**Additional file 1:** **Supplementary figures: Fig. S1.** Recursive feature elimination. Fig. S2. The nomogram prediction model based on multi-factor Logistic regression analysis. **Supplementary table: Table S1**. Performance comparison between XGBoost model and nomogram.

## Data Availability

The datasets used and/or analyzed during the current study are available from the corresponding author on reasonable request.
