# Supplementary material for: Prediction of nonsentinel lymph node metastasis in breast cancer patients based on machine learning
Source: World J Surg Oncol. 2023 Aug 11;21:244. doi: 10.1186/s12957-023-03109-3 (PMC10416453; doi:10.1186/s12957-023-03109-3)

| **Table S1 Performance comparison between XGBoost model and nomogram.** | | |
| --- | --- | --- |
| **Model** | **Training Set** | **Test Set** |
|  | AUC | AUC |
| **XGBoost** | 0.781 | 0.764 |
| **Nomogram** | 0.647 | 0.706 |


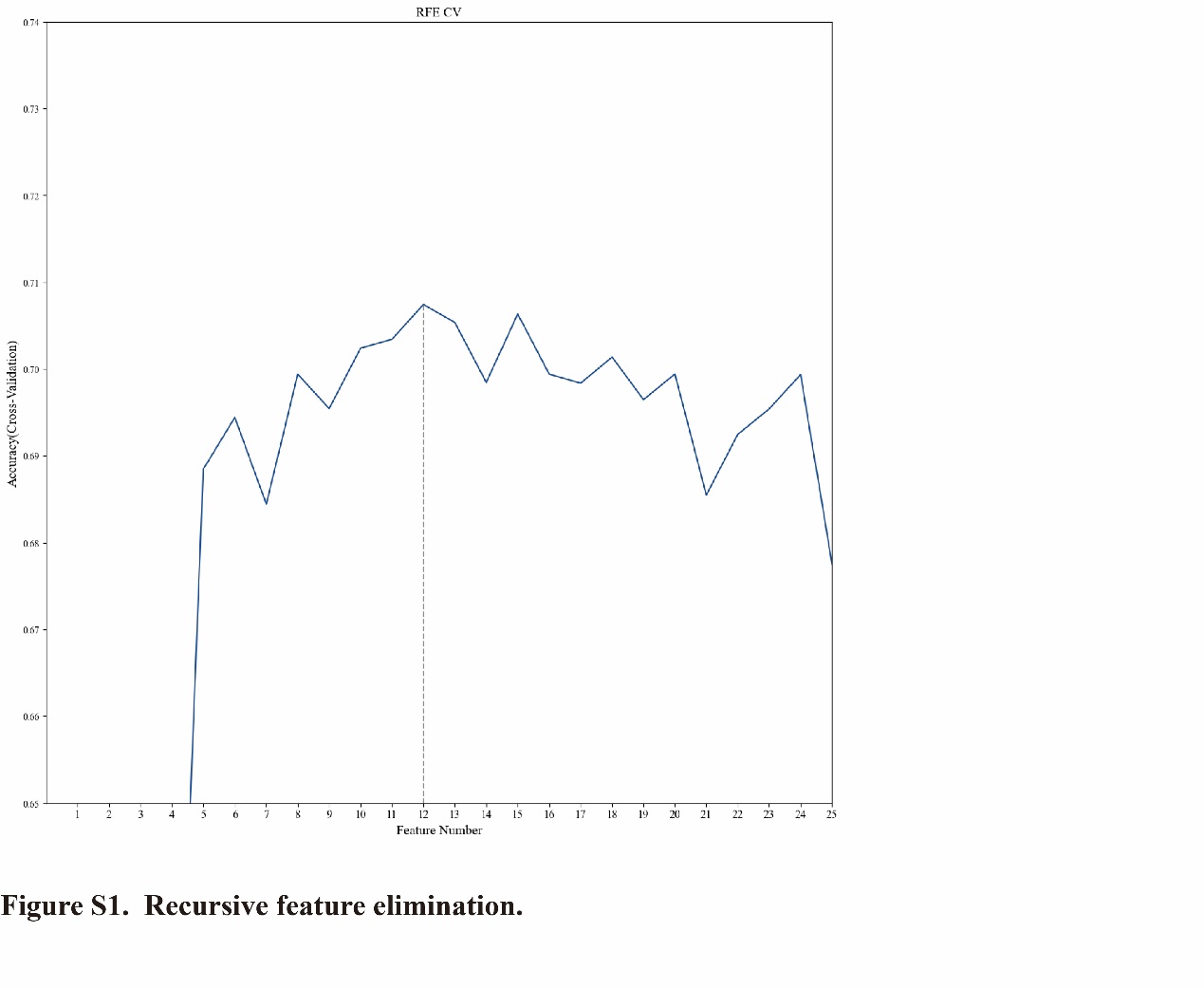


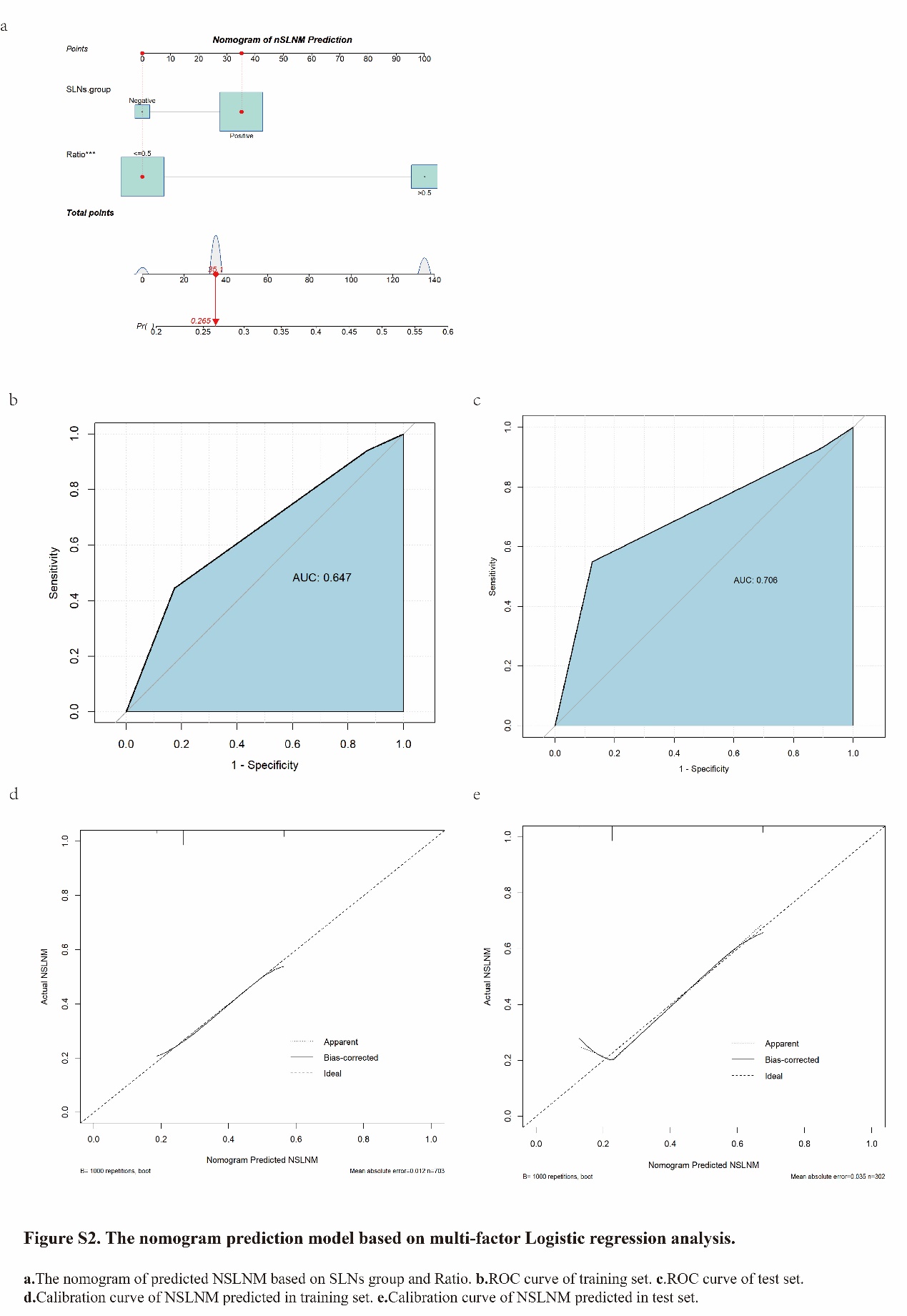

Supplement: Supplementary file 1 — Additional file 1: Supplementary figures: Fig. S1. Recursive feature elimination. Fig. S2. The nomogram prediction model based on multi-factor Logistic regression analysis. Supplementary table: Table S1. Performance comparison between XGBoost model and nomogram. [file 12957_2023_3109_MOESM1_ESM.docx]
